# Transactions of the Macon Medical Association

**Published:** 1871-05

**Authors:** 


### TRANSACTIONS OF THE MACON MEDICAL ASSOCIATION.

MACON, GA., March 7, 1871.

The Association was called to order by its President, Dr. Wm. F. Holt. The order of business having been concluded to oral communications, Dr. P. H. Wright reported a case of traumatic aneurism of the temporal artery resulting from a blow by a pebble or stone thrown from a distance, inflicting a contused wound in the temple. Previous to seeing the case, some ten days after its occurrence, another physician had been called in, and mistaking the tumor for an ordinary abscess, proceeded to open accordingly. This operation was followed by considerable hemorrhage, which was with difficulty arrested without taking up the artery. The incision healed, but set up an ulcerative process which obtained when Dr. Wright saw the case. After ineffectual efforts to relieve the patient by pressure and other local means, the trouble was overcome by passing a ligature around the artery. The difficulty of ligation was very great, and did not succeed until the needle was passed deep beneath the tissue, taking in a portion of the parotid gland, and including the whole within the loop. This entirely arrested the hemorrhage, and the clot being thoroughly turned out, the wound healed, and the patient made a speedy and perfect recovery. During the discussion, Dr. Hammond stated that he had seen a similar case, in which it was necessary to take up all the neighboring arterial branches to stop the bleeding.

Dr. D. W. Hammond reported a case of genuine cerebro-spinal meningitis, cured, as he believes, by the administration of hydrate of chloral, in 20-grain doses *pro re nata*. Very little other medicine was given; the chloral controlling all nervous symptoms perfectly.

Dr. C. B. Nottingham exhibited a small cellular polypus, excised from the neck of the uterus. He did not present the tumor for anything peculiarly striking in its appearance or character, but as it had well nigh proved the outlet to the woman's life, he thought a brief history of the case might not be uninteresting to the Association. The patient had given birth to two children, in due course of time following her miscarriage, but could not account for her sterility during the six years from the birth of her second child, to the death of her husband. She at one time supposed herself to be pregnant, and made the usual preparations, but only to be disappointed. She suffered greatly from Dysmenorrhœa. At times the flow was suppressed, and again in excess and very painful. She had been frequently importuued by her physician as to the propriety of submitting to a digital and specular examination; but a sense of *true* female delicacy denied him this important privilege until the excessive loss of blood, on one occasion, resulted in swooning, in which condition he found her on reaching the bedside, when he immediately proceeded to make a vaginal exploration. The examination detected the mass just without the os—the peduncle being small, and attached high up to the endotrachelium. A tampon was immediately applied to remain until the necessary steps could be taken to remove the polypus, which was done by introducing a Sims' speculum, drawing down the tumor, and excising at its attachment. The hemorrhage was controlled by persulphate of iron applied over pledgets of lint. No further trouble ensued, and recovery was prompt and satisfactory.

Dr. Nottingham accounts for the dysmenorrhœa and sterility in the occlusion of the cervical canal by the polypoid mass—the occupancy of which, in the neck, was a complete bar to the exit of the monthly flow, as well as to seminal transmission. The speculum exhibited a blood vessel of considerable size on its cut surface, by means of which the growth had been fed, and which was the cause of the little short of fatal hemorrhage.

